# A Whole-Cell System Based on Engineered Bacteria to Assess Cobalt Presence in Food: The Example of the Pasta Production Chain

**DOI:** 10.3390/bios15110763

**Published:** 2025-11-17

**Authors:** Monica De Caroli, Sara Carrozzo, Carla Perrotta, Patrizia Rampino

**Affiliations:** 1 Department of Biological and Environmental Sciences and Technologies, University of Salento, Via Monteroni 165, 73100 Lecce, Italy; monica.decaroli@unisalento.it (M.D.C.); sara.carrozzo@unisalento.it (S.C.);; 2 NBFC National Biodiversity Future Center, 90133 Palermo, Italy

**Keywords:** cobalt, engineered bacteria, food safety, pasta chain, *UspA* promoter, whole-cell biosensor

## Abstract

With the aim of developing a new tool to meet the increasing demand for food safety, a whole-cell-based system able to detect the presence of cobalt contamination along the pasta production chain was constructed. This system is based on bacterial cells engineered with a plasmid containing the *eGFP* gene under the control of a promoter sequence, and is able to elicit a fluorescence signal when activated. The promoters of four stress-responsive genes (*DnaK*, *GroE*, *UspA*, and *ZntA*) were used to test their responsiveness to cobalt; the promoter of the *UspA* gene, coding for a universal stress protein, was chosen. The *UspA* promoter was activated by cobalt, and the system described was highly sensitive, successfully detecting low concentrations of cobalt within complex food matrices derived from durum wheat seeds when exogenous cobalt was added. In food matrices tested alone, a fluorescence signal was present only in bran and fine bran, confirming that these parts of the wheat seed are the ones in which contaminants accumulate. Conversely, in the other matrices derived from the inner part of grains, no signal was detected. The findings reported contribute to the development a new, effective and sensitive tool for monitoring cobalt contamination, offering a valuable approach to enhance food safety control.

## 1. Introduction

Interest in food safety has significantly grown over the last few decades. Particular attention has been given to the potential risks to human health posed by the presence of contaminants in foods. In fact, the safety of food chains is often unbalanced by the presence of biological agents and/or chemical elements in excess amounts, potentially acting as a source of foodborne diseases.

The biological hazard derives mainly from microorganisms or from their metabolites, such as toxins. Chemical hazards can derive from food components, acting as allergens or toxins, as well as from environmental contaminants, such as antibiotics, heavy metals, etc.

Heavy metals, the most dangerous environmental contaminants, can derive from human activities, such as industrialization and massive use of fertilizer, which are responsible for the wide distribution of these elements in soils. Heavy metals reach the human body through water and food, constituting a severe threat to human health [1]. Their high metal ion toxicity is due to their non-biodegradable properties and their long biological half-life; as a consequence, these contaminants tend to accumulate in different human organs, often interfering with hormonal activity (they are among the potential causes of infertility), or causing general irritation, and cardiovascular and respiratory problems, leading to the development of severe pathologies or even death [2,3].

Heavy metal contamination can also derive from the food production chain and packaging processes. For instance, the durum wheat chain, spanning from grains to pasta, is a potential source of metal contamination due to the release of metal ions from the equipment used to transform grains into semola (milling), flour and pasta [4].

To meet the increasing demand for food safety and quality, the presence and concentration of heavy metals in food products have to be monitored. For this purpose, it is crucial to identify or generate rapid and accurate systems to monitor the presence of these contaminants in foods and throughout the entire food production chain. Traditionally, the determination of the presence of heavy metals is carried out using complex and expensive techniques, such as nuclear magnetic resonance spectroscopy or NMR, ion chromatography or IC, atomic absorption spectrometry or AAS, etc. However, all of these methods do not provide information regarding the bioavailability of these elements, on their effects on living organisms and on the synergistic/antagonistic effect that they can have when present in the body together with other substances. Hence, it is necessary to identify alternative methods to carry out heavy metal determination. In recent years, simpler and cheaper methods have been developed, to detect not only the presence of metal ions, but also their bioavailability [5,6]. Among these, biosensors have emerged as promising tools characterized by high sensitivity, rapidity, low cost, portability, ease of use and accuracy. Due to all these qualities, biosensors have become particularly interesting as analytical tools in environmental monitoring, medicine, agriculture, and, particularly, in food analysis [5,7,8,9]. Particularly relevant are cell-based biosensors, i.e., devices using living cells (acting as sensors) to detect the presence of analytes in biological samples, coupled with a transducer element converting a biological response into an easily measurable signal. The intrinsic characteristics of cell-based biosensors make them particularly suitable for preliminary screenings of food samples. Most whole-cell biosensors utilize bacterial cells because they are easy to be genetically engineered and are able to survive in adverse environmental conditions.

This work reports on our investigations into the ability of engineered bacteria to detect the presence of cobalt (Co) in food matrices derived from durum wheat grains. Cobalt is an essential trace element for both animals and humans, due to its key role in many biological processes. This element is involved in cell mitosis, the synthesis of neurotransmitters, the formation of myelin sheaths, the synthesis of vitamin B_12_, and the formation of red blood cells, since Co salts stimulate erythropoietin synthesis. However, at high concentrations, this element can cause some health issues, such as hair loss, heart diseases, thyroid damages, nausea, diarrhea, low blood pressure, etc. Moreover, Co has been classified as a Group2B carcinogen by the International Agency for Research on Cancer (IARC), indicating its possible carcinogenicity in humans [10]. To set up a whole-cell system that is able to detect Co presence, engineered bacteria cells were used. These cells contained a plasmid carrying the *eGFP* gene under the control of the promoter sequence of the bacterial *UspA* gene. This gene encodes a universal stress protein, and its promoter has been revealed to be activated by the presence of Co. The system described here demonstrates the ability to detect low concentrations of Co in food matrices too. The findings presented in this study contribute to the development of a novel tool that is useful for food safety monitoring.

## 2. Materials and Methods

### 2.1. Bacterial Cells and Media

The cells used were of *Escherichia coli* K12 strain MG1655 transformed with pMS201, a low-copy plasmid used to clone and characterize *E. coli* promoters. In particular, these cells belong to a collection of clones distinguished by the presence of plasmids carrying a gene coding for a fast-folding GFP fused to the full-length sequence of an *E. coli* promoter. These plasmids allow us to measure the expression of the *eGFP* gene at high time resolutions in an accurate and reproducible way [11]. Four clones were selected in this study, carrying p*DnaK-eGFP*, p*GroE-eGFP*, p*UspA-eGFP*, and p*ZntA*-*eGFP*, each containing the promoter region of *DnaK*, *GroE*, *UspA* and *ZntA* genes, respectively. All clones were purchased from Thermo Scientific (Waltham, MA, USA).

Cells were grown in 20 mL of liquid 2X LB medium-low salt (Thermo Scientific) with added peptone (10 g/L, Thermo Scientific), yeast extract (5 g/L, Thermo Scientific) and kanamycin (25 mg/L Thermo Scientific) to the optical density at 600 nm (OD_600_) = 0.6. Cells were then harvested and resuspended either in liquid medium or immobilized in agarose and Na alginate matrices.

### 2.2. Immobilization Matrice Preparation

Cells were immobilized using two different matrices, namely agarose and Na alginate.

The agarose immobilization matrix was prepared as follows: 0.6 g of LB (Thermo Scientific) and 0.4 g of Agarose (Thermo Scientific) were added to 20 mL of sterile distilled water; the medium was autoclaved for 16 min and subject to a temperature of 40 °C before mixing it with the cells resuspended in 0.5 mL of 2X LB. The mix, made of the agarose immobilization matrix and transformed cells, was aliquoted in a 96 multi-well plate (150 μL for well).

The Na alginate immobilization matrix was prepared in accordance with Köler et al. [12]. The cells, grown as reported above, were pelleted and washed with 0.9% NaCl Sigma-Aldrich, St. Louis, MO, USA). The cells were then resuspended in 2.5 mL of 0.9% NaCl and 2.5 mL of 4% Na alginate (Sigma-Aldrich), the cell suspension was distributed in a 96 multi-well plate (75 μL for well). To induce gelation, 50 μL of 0.1 M CaCl_2_ (Sigma-Aldrich) was added to each well; after 10 s, 50 μL of TRIS acetate (Sigma-Aldrich) 40 mM pH 7 was added.

### 2.3. Induction Assay

The assay was performed on the bacterial cells, starting from three independent colonies grown overnight in 2X LB medium -low salt (Thermo Scientific) supplemented with peptone (10 g/L, Thermo Scientific), yeast extract (5 g/L, Thermo Scientific) and kanamycin (25 mg/L Thermo Scientific). The cells were then diluted 150-fold in fresh 2X LB medium and regrown to OD_600_ = 0.6.

To evaluate the bacterial promoters’ activity, the cells, either in liquid medium or immobilized, were subjected to different stress conditions, challenging them with CoCl_2_ (Sigma-Aldrich) at different concentrations and for different times, as indicated for each experiment.

Stress in the liquid medium was applied on bacteria transformed with the four plasmids, grown as reported above, and subjected to treatment with CoCl_2_ in amounts from 0.005 μM to 25 μM for 1, 2, 3 and 4 h.

Stress on the immobilized cells was applied only on bacteria transformed with p*UspA-eGFP*. Stress treatments were performed by adding CoCl_2_, CdSO_4_ (Sigma-Aldrich), NiCl_2_ (Sigma-Aldrich) or ZnCl_2_ (Sigma-Aldrich), in amounts from 0.0001 μM to 1 μM, to each well for 1, 2, 3 and 4 h.

### 2.4. Fluorescence Determination

Fluorescence was measured using an InfiniteF200 fluorometer (TECAN, Männedorf, Switzerland), with the following settings: excitation: 485 nm (±20); emission: 510–560 nm. Fluorescence values were presented as arbitrary fluorescence units (FUs) or as relative fluorescence units (RFUs), the latter calculated as the ratio of the fluorescence of the treated sample to that of the untreated control. All the independent experiments were carried out in triplicate and repeated at least two times.

Transformed bacterial cells were subjected to further fluorescence quantification using a laser scanning confocal microscope (LSM 710, Zeiss, Jena, Germany). A 488 nm argon ion laser line was utilized to detect GFP fluorescence, and a 505–530 nm filter set was used to record the emission. To ensure comparability across experiments, the laser line power, gain, and offset settings were kept constant for each experiment. The Profile Tool of the ZEN2012 program (Black edition, version 8) of the LSM 710 confocal microscope was used to quantify the GFP fluorescence. Fluorescence quantification was performed across three independent experiments, evaluating at least 10 transformed bacterial cells per treatment. Image assembly was carried out using Adobe Photoshop 7.0 (Mountain View, CA, USA).

### 2.5. Food Matrices Utilized for Co Detection

Food matrices, utilized to assess the responsiveness of the engineered bacteria to the presence of Co, were derived from durum wheat. These included bran, fine bran, semolina, flour and pasta. An amount of 2 g of each food matrix weas added to 8 mL of sterile distilled water, and the mixture was stirred for 2 h. Then, 1 mL of the resulting suspension was centrifuged at 13,000 rpm for 2 min and 50 μL of the supernatant was added to the immobilized bacteria.

### 2.6. Statistical Analysis

Statistical analysis was performed using SigmaStat version 3.11 software (Systat Software Inc., Chicago, IL, USA). All data are presented as the mean of at least three different measurements ± standard deviation (SD), and were analyzed by ANOVA followed by Tukey’s pairwise post hoc test. A *p* value ≤ 0.05 was considered statistically significant.

## 3. Results

### 3.1. Evaluation of Engineered E. coli Cells Responsiveness to Co

Genetically modified *E. coli* cells, carrying the plasmid with the promoter activated by the presence of Co fused to the *eGFP* reporter gene, were used to detect the presence of Co at low concentrations. Four *E. coli* genes that were particularly suitable for this purpose were identified, including *DnaK* and *GroE*, both encoding heat shock proteins produced in response to heat stress, *ZntA*, encoding an ATPase involved in the efflux of zinc, cadmium, lead, nickel and cobalt ions, and *UspA*, encoding the uspA protein, produced in response to a wide number of different environmental stresses [13]. The promoters of these genes are sensitive to many factors, including metal ions. Using an *E. coli* promoter collection [11], four different clones were isolated containing the p*DnaK-eGFP*, p*GroE-eGFP*, p*UspA-eGFP* and p*ZntA-eGFP* plasmids, carrying the *DnaK*, *GroE*, *UspA* and *ZntA* promoters, respectively, fused to the *eGFP* gene. These clones were resuspended in liquid medium and tested against CoCl_2_ at different concentrations (0.5 μM, 1 μM and 10 μM) for 2 h.

**Figure 1:**
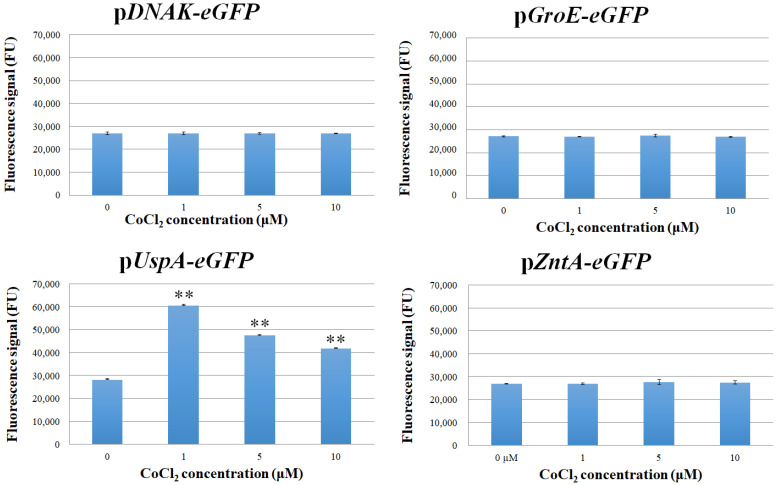
Fluorescence signal of *E. coli* cells transformed with *pDNAK-eGFP*, pGroE-eGFP, *pUspA-eGFP*, and *pZntA-eGFP* after treatment with CoCl_2_ for 2 h, in liquid medium. Each value corresponds to the mean of at least three independent measurements ± S.D. The asterisks indicate that the values reflected in the data are significantly higher than those in the control data (Tukey’s post hoc test, ** *p* < 0.01). FU = fluorescence unit.

Changes in the production level of eGFP were detected using a fluorometer, and the results obtained, reported in Figure 1, indicate that, except for the clone carrying the p*UspA-eGFP* promoter, there were no significant changes in the *eGFP* expression level following exposure to the different CoCl_2_ concentrations. In contrast, cells harboring p*UspA-eGFP* exhibited a clear response: fluorescence intensity signals, when the CoCl_2_ concentration varied from 1 to 10 μM, were always higher, reaching the maximum at 1 μM and decreasing when the tested concentration were 5 and 10 μM.

**Figure 2:**
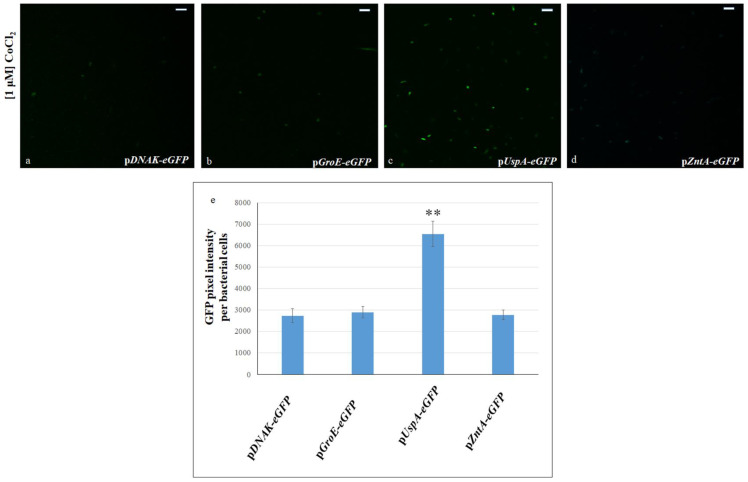
Confocal microscopy images of *E. coli* cells expressing GFP under the control of different stress-responsive promoters. Bacterial cells were transformed with p*DNAK-eGFP* (**a**), p*GroE-GFP* (**b**), p*UspA-eGFP* (**c**) and p*ZntA-eGFP* (**d**), and treated with 1 μM CoCl_2_. Scale bars: 20 μm. Objective: 40×; zoom: 0.6×. (**e**) Quantification of fluorescence intensity, expressed as the mean of GFP pixel intensity per transformed bacterial cell, after treatment with 1 μM CoCl_2_. Bacterial cells transformed with p*UspA-eGFP* exhibited a significantly higher fluorescence signal compared to those transformed with the other constructs. The asterisks indicate that the values reflected in the data are significantly higher than those in the control data (Tukey’s post hoc test, ** *p* < 0.01).

Visual confirmation of eGFP production was obtained by observing the transformed bacterial cells under a confocal microscope. Cells harboring the *UspA-eGFP* plasmid exhibited a marked increase in GFP fluorescence intensity compared to cells carrying the other plasmids, as further confirmed by quantitative measurement of the GFP signal intensity (Figure 2).

Based on these results, the p*UspA-eGFP* clone was selected for further investigations.

### 3.2. Evaluation and Optimization of E. coli Cell-Based Co Detection System

**Figure 3:**
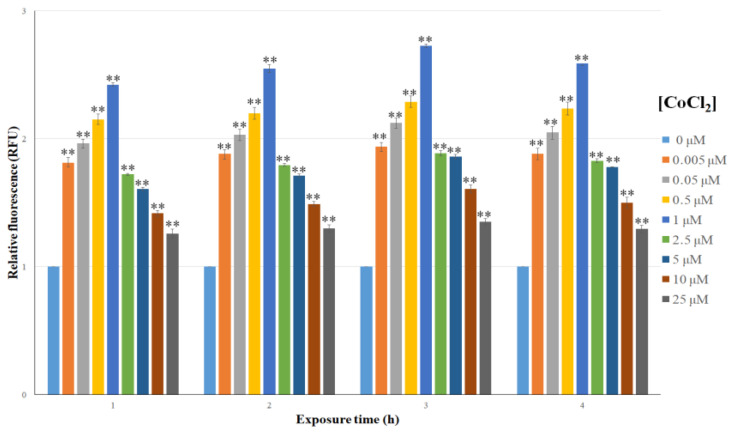
Relative fluorescence of *E. coli* cells transformed with p*UspA-eGFP*, after treatment with different CoCl_2_ concentrations, evaluated after 1, 2, 3, and 4 h of exposure, in liquid medium. Each value corresponds to at least the mean of three independent measurements ± S.D. The asterisks indicate that the values in the data are significantly higher than those in the control data (Tukey’s post hoc test, ** *p* < 0.01). RFU = relative fluorescence unit.

To optimize the experimental conditions in which the engineered bacteria sense the presence of Co, *E. coli* cells harboring the p*UspA-eGFP* plasmid were exposed to 0–25 μM CoCl_2_ in liquid medium, and the fluorescence intensity was measured from 1 to 4 h. Changes in fluorescence signals were expressed as RFUs. The results reported in Figure 3 indicate that all the tested concentrations induced *UspA* promoter activity, resulting in increased GFP production. Fluorescence intensity increased as the CoCl_2_ concentration varied from 0.005 to 1 μM, and then decreased as the CoCl_2_ concentration varied from 2.5 to 25 μM. At all of the tested concentrations, the fluorescence intensity was observed after 1 h and reached the maximum value (>2.5 fold) after 3 h with 1 μM CoCl_2_.

**Figure 4:**
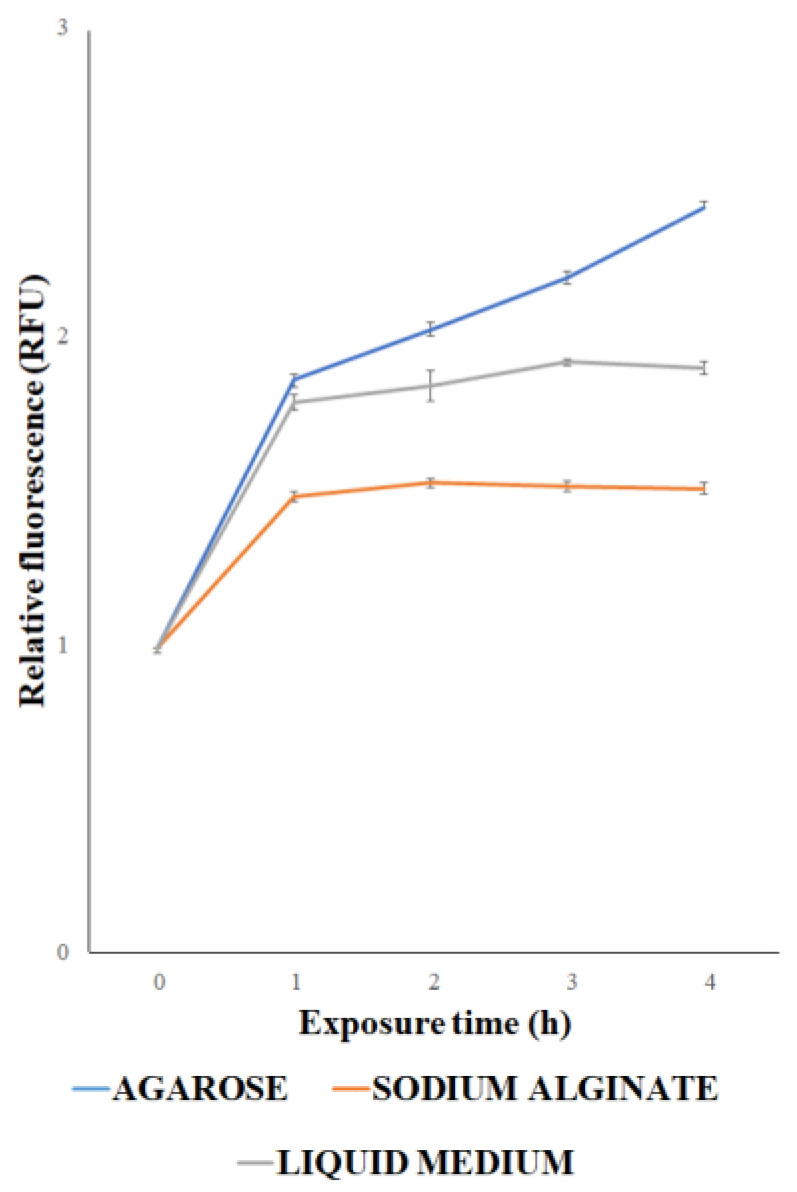
Relative fluorescence of *E. coli* cells transformed with p*UspA-eGFP*, floating in liquid medium, immobilized in agarose, or immobilized in Na alginate, after treatment with CoCl_2_ 1 μM for 1, 2, 3 and 4 h. Each value corresponds to the mean of at least three independent measurements ± S.D. RFU = relative fluorescence unit.

Once we had identified the best induction conditions, the *E. coli* cells, at the same concentration, were immobilized using two different matrices (agarose and Na alginate). GFP production was assayed in the presence of 1 μM CoCl_2_ over different time points (1 to 4 h). The assay was also performed on cells, at the same concentration, floating in liquid medium. The results shown in Figure 4 indicate that the fluorescence signal was already detectable after 1 h in all the assays. However, both cells immobilized in the Na alginate matrix and floating in liquid medium exhibited lower fluorescence compared to agarose-immobilized cells at all time points. In these two conditions, fluorescence reached a maximum after 1 h and remained nearly constant until 4 h. On the contrary, agarose-immobilized cells exhibited a fluorescence signal increasing until 4 h, which awas always higher compared to that of Na alginate-immobilized *E. coli* cells. Based on these data, agarose was chosen as the preferred immobilization matrix.

**Figure 5:**
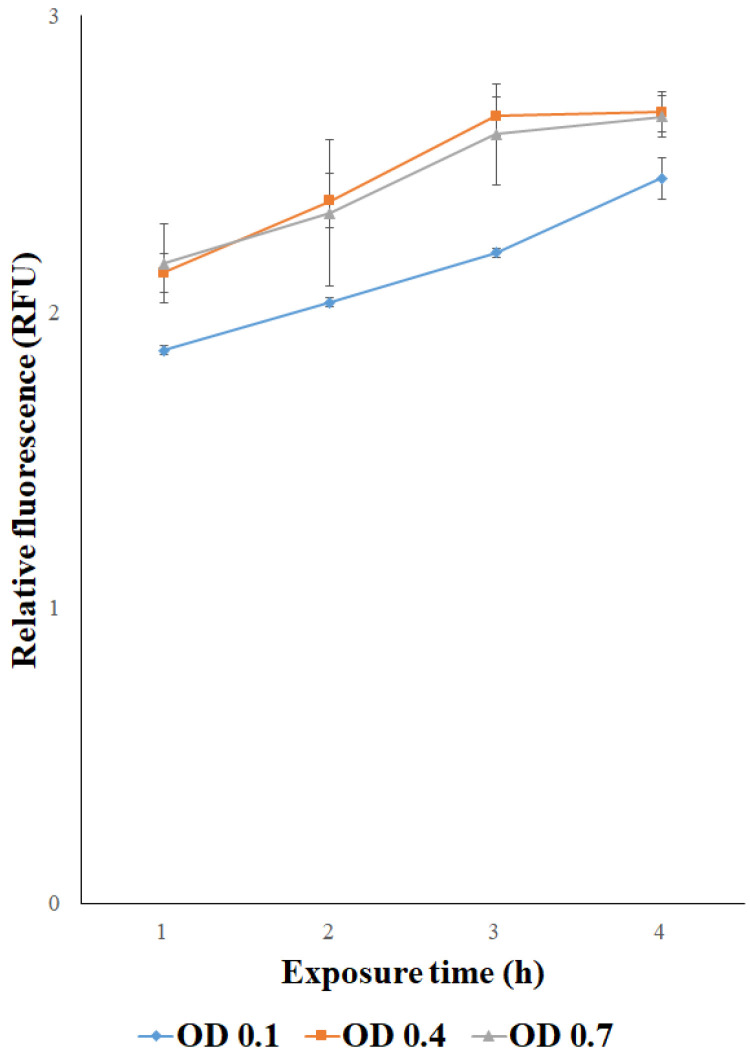
Relative fluorescence of *E. coli* cells transformed with p*UspA-eGFP*, immobilized in agarose at three different cell concentrations (OD = 0.1, OD = 0.4, OD = 0.7), treated with CoCl_2_ 1 μM. Each value corresponds to the mean of at least three independent measurements ± S.D. RFU = relative fluorescence unit.

To optimize this sensor system, further characterization was performed to determine the best cells’ concentration. Three different cell concentrations (OD = 0.1, OD = 0.4, OD = 0.7) were used to study the variation in fluorescence signal in response to 1 μM CoCl_2_ treatment for 1, 2, 3, and 4 h. As shown in Figure 5, the highest fluorescence signal was observed when immobilized cells were at OD = 0.4 or OD = 0.7 across all time points. In contrast, immobilized cells at OD = 0.1 always exhibited a lower fluorescence signal. Based on these results, OD = 0.4 was used for further determinations.

### 3.3. Analysis of Sensitivity of E. coli Cell-Based Detection System to Co and Other Metals

**Figure 6:**
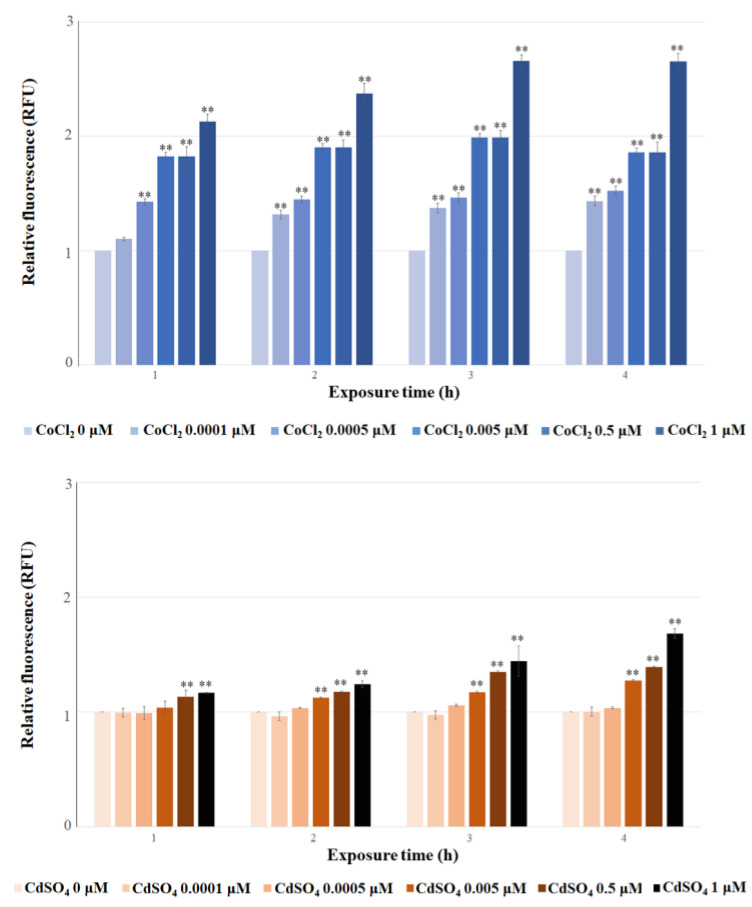

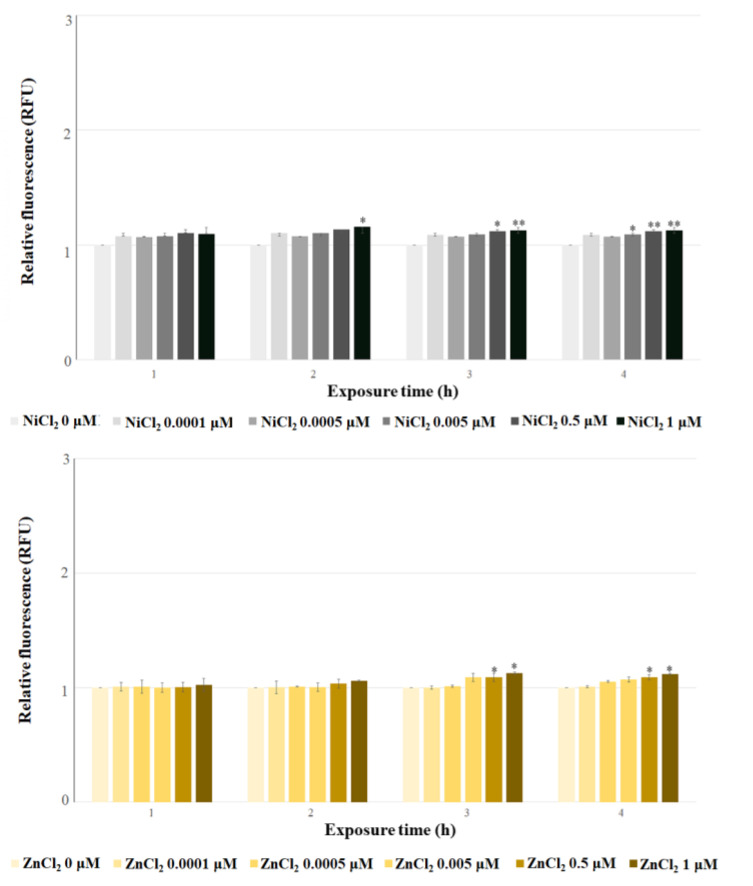
Relative fluorescence of *E. coli* cells transformed with p*UspA-eGFP*, immobilized in agarose, treated with different concentrations of CoCl_2,_ CdSO_4_, NiCl_2_, and ZnCl_2_ (0–1 μM), evaluated after 1, 2, 3, and 4 h of exposure. Each value corresponds to the mean of at least three independent measurements ± S.D. The asterisks indicate that the values in the data are significantly higher than those in the control data (Tukey’s post hoc test, * *p* < 0.05; ** *p* < 0.01). RFU = relative fluorescence unit.

Once the cell-based Co detection system was optimized with regard to the immobilization matrix and cell concentration, its ability to elicit *eGFP* expression at different CoCl_2_ concentrations was verified. The detection system was challenged against CoCl_2_ from 0.0001 to 1 μM, and the fluorescence signal was registered after 1, 2, 3 and 4 h. The results obtained and reported in Figure 6 indicate that the GFP level increased from 0.0001 to 1 μM CoCl_2_. Notably, fluorescence induction was detectable after just 1 h of treatment. The data reported indicate that the optimized cell-based system can detect the presence of CoCl_2_ at very low concentrations, down to 0.0001 and 0.0005 μM.

To assess the sensitivity of the optimized cell-based system to other metals contaminating the environment, this system was tested against the same concentration (from 0.0001 to 1 μM) of CdSO_4_, NiCl_2_ and ZnCl_2_. In general, fluorescence was elicited by all the utilized salts at lower levels compared to CoCl_2_ (Figure 6). For CdSO_4_, fluorescence was first detectable after 1 h at 0.5 and 1 μM and after 2 h at 0.005 μM. For NiCl_2_, the first signal appeared after 2 h at 1 μM, after 3 h at 0.5 and 1 μM, and after 4 h, from 0.005 μM onwards. For ZnCl_2_, a detectable response occurred after 3–4 h at 0.5 and 1 μM concentrations. These results indicate that the sensitivity of this system to the tested metal salts is consistently lower than its sensitivity to Co.

### 3.4. Applicability of E. coli Cell-Based Co Detection System to Food Analysis

**Figure 7:**
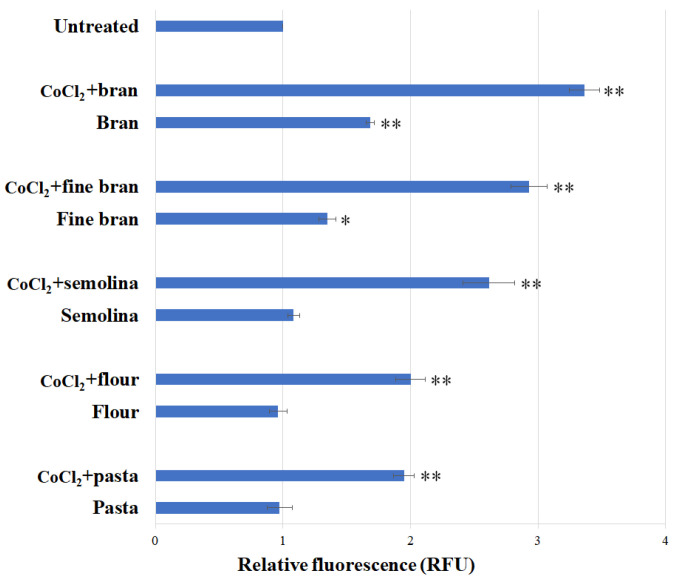
Relative fluorescence of *E. coli* cells transformed with p*UspA-eGFP*, immobilized in agarose (cell concentration OD = 0.4), with the addition of different food matrices (in the presence or absence of 1 μM of CoCl_2_) for 2 h. Each value corresponds to the mean of at least three independent measurements ± S.D. The asterisks indicate that the values in the data are significantly higher than those in the control data (Tukey’s post hoc test, * *p* < 0.05, ** *p* < 0.01). RFU = relative fluorescence unit.

To evaluate the ability of the optimized *E. coli* cell-based system to sense Co contamination in food, this system was tested on artificially contaminated food matrices with added CoCl_2_ to a final concentration of 0.5 μM. In particular, our focus was on evaluating Co contamination along the pasta chain. Accordingly, bran, fine bran, semolina, durum wheat flour and pasta were analyzed to check each step of the pasta production process, from milling to the final product. As shown in Figure 7, fluorescence signals were elicited in all artificially contaminated samples; the highest signals were detected in bran and fine bran. The *E. coli*-based system was subsequently tested on the food matrices without added CoCl_2_. A statistically significant increase in fluorescence was detected only in bran (RFU about 1.7) and fine bran (RFU about 1.3), indicating a detectable level of GFP expression in these samples.

## 4. Discussion

Heavy metals released from industrial activities contaminate the environment and may pose serious risks to human health. Cobalt is a so-called transition metal positioned between iron and nickel in the periodic table, and it is one of the trace elements naturally present in air, water, and soil [10,14]. Anthropogenic activities such as mining, manufacturing and agriculture have significantly increased its environmental levels. Cobalt is an essential trace element for humans; however, harmful properties in this element, derived from its potential accumulation in the food chain, can cause damage to animals and humans, leading to different disorders [15,16]. Monitoring of this element is not as strict as it is for dangerous elements due to its wide distribution in nature; nevertheless, its detection in food is necessary, since high intake of Co is toxic. In this context, it is of paramount importance to set up a highly sensitive and user-friendly method for Co determination, allowing for the rapid monitoring of its presence in food. Conventional analytical techniques for trace element determination are often time-consuming and expensive, and they fail to provide information about metal bioavailability. In recent years, simpler and cheaper methods have been developed to detect not only the presence of metal ions but also their bioavailability; some of them are based on engineered whole cells that are able to sense the presence of metal ions [5,6].

In this paper, we report the establishment of an *E. coli* cell-based system that is able to rapidly and efficiently detect the presence of Co in food matrices. This method was chosen considering that cells, acting as a biorecognition element in biosensors, can target diverse chemicals, antibiotics and heavy metals, assessing food quality and safety in accordance with the most recent requirements of food analysis. The genetic system on which they are based relies on the modification of reporter gene expression triggered by the activation of specific stimulus-responsive promoters [17]. The whole-cell system here reported is based on the ability of *E. coli* cells to exhibit variations in the expression of the *eGFP* gene in response to an external stimulus. Thus, a few promoters, known to be activated under different stress conditions, were tested to identify the one most sensitive to Co presence. Among these, the promoter of the *UspA* gene exhibited the strongest response in liquid culture medium. *UspaA* belongs to a gene family that is widely distributed in living organisms and essential for their survival under adverse conditions [18,19]. The *UspA* promoter is responsive to multiple stimuli, including nutrient starvation, exposure to toxics, and other adverse environmental conditions [13]; our attention was focused on its responsiveness to metal ions. Bacterial cells, engineered with a plasmid containing the e*GFP* gene under the control of the *UspA* promoter, were used. They were tested at different CoCl_2_ concentrations in liquid medium, with their fluorescence due to GFP production being measured. This allowed for the identification of the CoCl_2_ concentration corresponding to the best induction conditions. The whole-cell system parameters were then optimized, testing *E. coli* cells’ ability to sense low concentrations of CoCl_2_ using two different immobilization matrices and various cell concentrations. Cell immobilization is important for obtaining a stable and reliable system suitable for on-site applications. Moreover, it is a good tool with which to obtain bacterial cells confined in a space region that can be used repeatedly and continuously [20,21].

The developed *E. coli* cell-based system was also tested using salts of other metals known to be present as contaminants in soils. According to the data reported here, cobalt induced fluorescence in the whole-cell system at much lower concentrations than any of the other metals tested. In general, the other contaminating metals elicited a detectable response only at higher concentrations and after longer exposure times. Given the high sensitivity of the system toward low cobalt concentrations, it was applied to various food matrices to verify its ability to detect the presence of Co in food.

The main focus of this research was on one of the most commonly utilized crops worldwide: durum wheat. Durum wheat is a crop widely employed for human consumption; it is the main source of carbohydrates and thus of energy for humans. However, durum wheat grains often contain contaminants, including metals present in the environment [22]. One of these contaminants is cobalt; it is known that Co accumulates in various parts of the plant, including the grain [23]. To test the applicability of the whole-cell system to durum wheat grains, we tested different food matrices related to the steps of the durum wheat chain, from grains to pasta; in particular, the matrices analyzed were bran, small bran, semolina, durum wheat flour, and pasta. As a first step, the ability of the whole-cell system to detect cobalt in these food matrices was evaluated by adding a known concentration of CoCl_2_ to each sample. The data obtained indicate that the peculiar characteristics of this device are i) its ability to sense very low Co concentrations and ii) its capacity to maintain this sensitivity in the presence of complex food matrices that could exert a “quenching” effect. Having assessed its applicability, the device was tested against the food matrices without added cobalt. The data obtained revealed that a fluorescence signal was elicited only by two samples, i.e., fine bran and the bran fraction. Grains of durum wheat are characterized by the presence of an external outer layer, called bran, which is typically a by-product of milling, with both food and non-food applications [24,25]. Over the years, the use of wheat bran for human consumption has increased due to its high content of minerals, vitamins, and bioactive compounds, which have health-promoting properties. Studies performed during the last few years indicate that whole-grain products contain higher levels of contaminants compared to refined products. According to the data reported in this work, a fluorescence signal was detectable in bran and fine bran, confirming that these parts of the wheat seed are the ones in which contaminants accumulate. Conversely, the other food matrices examined, all deriving from the inner part of grains, did not elicit fluorescence; in fact, it is well known that refining grains removes up to 80% of polynutrients from whole grains, but also reduces the presence of many contaminants [22].

## 5. Conclusions

The findings of this study indicate that the *E. coli* cell-based system described here is rapid, sensitive and easy to use, and can be applied to complex food matrices. To our knowledge, this is, so far, the first example of a device based on engineered *E. coli* cells that is able to detect Co presence in samples related to the pasta production chain. This was demonstrated by detectable fluorescence signal when the system was challenged with food matrices following the addition of exogenous CoCl_2_.

To fully realize the potential of this device, considering the complexity of different food matrices, in the future, further calibration and standardization will be necessary. These steps are essential for biosensor’s obtention of approval and validation for use in the food industry to ensure food safety.

## Data Availability

The original contributions presented in this study are included in the article.
